# Remote, real-time expert elicitation to determine the prior probability distribution for Bayesian sample size determination in international randomised controlled trials: Bronchiolitis in Infants Placebo Versus Epinephrine and Dexamethasone (BIPED) study

**DOI:** 10.1186/s13063-022-06240-w

**Published:** 2022-04-11

**Authors:** Jingxian Lan, Amy C. Plint, Stuart R. Dalziel, Terry P. Klassen, Martin Offringa, Anna Heath, on behalf of the Pediatric Emergency Research Canada (PERC) KIDSCAN/PREDICT BIPED Study Group

**Affiliations:** 1 Child Health Evaluative Sciences, The Hospital for Sick Children, 686 Bay Street, Toronto, ON M5G 0A4 Canada; 2 Division of Emergency Medicine, Children’s Hospital of Eastern Ontario, Ottawa, Canada; 3 Departments of Pediatrics and Emergency Medicine, University of Ottawa, Ottawa, Canada; 4 Children’s Hospital of Eastern Ontario Research Institute, Ottawa, Ontario Canada; 5 Departments of Surgery and Paediatrics: Child and Youth Health, University of Auckland, Auckland, New Zealand; 6 Children’s Emergency Department, Starship Children’s Hospital, Auckland, New Zealand; 7 University of Manitoba, Winnipeg, Manitoba Canada; 8 Children’s Hospital Research Institute of Manitoba, Winnipeg, Manitoba Canada; 9 Institute of Health Policy, Management and Evaluation, University of Toronto, Toronto, Ontario Canada; 10 Division of Neonatology, The Hospital for Sick Children, University of Toronto, Toronto, Ontario Canada; 11 Division of Biostatistics, Dalla Lana School of Public Health, University of Toronto, Toronto, Ontario Canada; 12 Department of Statistical Science, University College London, London, UK

**Keywords:** Expert elicitation, Bayesian statistics, Randomised controlled trials, Sample size determination, Prior probability distribution, Trial design

## Abstract

**Background:**

Bayesian methods are increasing in popularity in clinical research. The design of Bayesian clinical trials requires a prior distribution, which can be elicited from experts. In diseases with international differences in management, the elicitation exercise should recruit internationally, making a face-to-face elicitation session expensive and more logistically challenging. Thus, we used a remote, real-time elicitation exercise to construct prior distributions. These elicited distributions were then used to determine the sample size of the Bronchiolitis in Infants with Placebo Versus Epinephrine and Dexamethasone (BIPED) study, an international randomised controlled trial in the Pediatric Emergency Research Network (PERN). The BIPED study aims to determine whether the combination of epinephrine and dexamethasone, compared to placebo, is effective in reducing hospital admission for infants presenting with bronchiolitis to the emergency department.

**Methods:**

We developed a Web-based tool to support the elicitation of the probability of hospitalisation for infants with bronchiolitis. Experts participated in online workshops to specify their individual prior distributions, which were aggregated using the equal-weighted linear pooling method. Experts were then invited to provide their comments on the aggregated distribution. The average length criterion determined the BIPED sample size.

**Results:**

Fifteen paediatric emergency medicine clinicians from Canada, the USA, Australia and New Zealand participated in three workshops to provide their elicited prior distributions. The mean elicited probability of admission for infants with bronchiolitis was slightly lower for those receiving epinephrine and dexamethasone compared to supportive care in the aggregate distribution. There were substantial differences in the individual beliefs but limited differences between North America and Australasia. From this aggregate distribution, a sample size of 410 patients per arm results in an average 95% credible interval length of less than 9% and a relative predictive power of 90%.

**Conclusion:**

Remote, real-time expert elicitation is a feasible, useful and practical tool to determine a prior distribution for international randomised controlled trials. Bayesian methods can then determine the trial sample size using these elicited prior distributions. The ease and low cost of remote expert elicitation mean that this approach is suitable for future international randomised controlled trials.

**Trial registration:**

ClinicalTrials.gov NCT03567473

**Supplementary Information:**

The online version contains supplementary material available at 10.1186/s13063-022-06240-w.

## Background

Bayesian statistical methods use Bayes’ theorem to combine data with previous evidence, characterised in a prior distribution, to make inferences about the parameters in a statistical model [1]. Bayesian methods are increasingly popular in clinical research as concern about frequentist methods has increased [2]. Bayesian methods also formally incorporate external evidence into the trial conclusions, rather than making definitive conclusions based on a single trial [3]. Finally, they provide a more natural interpretation of uncertainty [4] and easily support frequent monitoring and adaptive designs [5].

To use Bayesian methods, the proposed trial sample size must be determined. Bayesian methods for sample size determination (SSD) have several advantages over frequentist methods. First, Bayesian SSD methods incorporate the statistical uncertainty that is inherent in the estimates of key quantities [6]. This contrasts to frequentist methods where the required sample size is highly sensitive to the fixed values that must be specified for several key quantities, such as size and the target difference [7, 8]. Secondly, frequentist SSD methods do not consider clinicians’ current beliefs about a treatment, meaning that trial results that contradict strong beliefs are often not convincing enough to change clinical practice [9]. Finally, sample sizes calculated using frequentist methods are often hard to achieve or unfeasible in rare diseases [10]. In this setting, Bayesian SSD methods can reduce the required sample size by combining trial data with other information, such as expert knowledge or earlier studies, to provide a similar level of scientific certainty [11].

To utilise Bayesian SSD methods, a “prior distribution” must be defined to represent the currently available evidence about all model parameters [12]. This prior distribution can be defined using historical data [13], expert knowledge or a combination of the two [8]. To use expert knowledge, it must be converted into a quantitative expression. This is commonly achieved through a structured “elicitation process” [14] in which experts are assisted in converting their knowledge into a distribution through a process that is viewed as formal data acquisition process based on validated methodologies [15].

Expert elicitation in clinical trials is becoming more frequent with 42 studies related to clinical trial design and analysis included in a recent review of 460 studies discussing Bayesian prior elicitation [16]. Elicitation has been used in randomised controlled trials (RCTs) that compare treatments for trauma resuscitation [17], bacterial corneal ulcers [18] and in rare diseases [19]. However, these studies required experts to meet in person, which can be difficult to arrange, extremely expensive, especially in international studies, and has been restricted due to the COVID-19 pandemic. Alternative approaches to in-person meetings are asynchronous surveys and exercises [20–22] or remote, real-time panels [23]. Asynchronous elicitation exercises often have low response and engagement rates and only allow for limited assistance during the elicitation session [22]. Furthermore, experts are less able to discuss and calibrate their beliefs [21], which is key to many elicitation frameworks [24, 25].

As RCTs aim to gather robust empirical evidence that could change clinical practice and health outcomes, the prior for the parameters in an international RCT should robustly represent the beliefs of experts in all health systems where the results would be implemented. This representation is particularly important in diseases where there are regional (international) differences in clinical practice and presentation patterns. Therefore, to avoid the restrictions of in-person exercises and the limitations of asynchronous elicitation, we implemented an efficient, remote, real-time elicitation process to generate representative priors to support Bayesian SSD.

Bronchiolitis, a viral infection of the small and medium airways, is the most common reason infants younger than 1 year of age are admitted to hospital in the developed world. Bronchiolitis also has strong regional differences in clinical practice [26]. Current management recommended by national guidelines is predominantly the provision of parenteral fluids for hydration and oxygen for hypoxemia, called “supportive care” [27–32]. Despite a lack of high-quality evidence, the use of additional pharmacotherapy such as nebulised epinephrine, albuterol, hypertonic saline or oral corticosteroids varies by region, with an odds of use of any of these of 11.5 in Canada and 6.8 in the USA, compared to Australia and New Zealand [26]. While using pharmacotherapy alongside supportive care is not supported by most guidelines, exploratory evidence suggests that the combination of inhaled epinephrine and oral corticosteroids has the potential to reduce hospital admission by a third in infants presenting to emergency departments (EDs) with bronchiolitis [33].

The Bronchiolitis in Infants with Placebo versus Epinephrine and Dexamethasone (BIPED) study is an international RCT comparing inhaled epinephrine and oral dexamethasone (a corticosteroid) to placebo for infants presenting to EDs with bronchiolitis for the primary outcome of reducing admission into hospital, taking place in Canada, New Zealand and Australia. Given the regional differences in bronchiolitis management and the geographical spread of BIPED sites, we describe our remote, real-time elicitation exercise to provide a well-justified, representative prior for the SSD and analysis of the BIPED study and the resulting Bayesian SSD.

## Methods

### The BIPED study

The BIPED study is a phase III, multi-centre, randomised, double-blind, placebo-controlled trial within the Pediatric Emergency Research Network (PERN) [34] to determine whether the combination of inhaled epinephrine and oral dexamethasone (EpiDex) reduces hospitalisation within the 7 days following an initial presentation to an ED with bronchiolitis. The BIPED study enrols participants across 12 international sites in the global network of networks PERN: six sites in Canada (part of the Pediatric Emergency Research Canada Network) and 3 in New Zealand and 3 in Australia (members of the PREDICT network). The study will enrol infants aged between 60 days and 1 year who present to the ED with an episode of wheezing or crackles, alongside signs of an upper respiratory tract infection during the peak season for respiratory syncytial virus (RSV). The active treatment, to be compared with a placebo control, is two treatments of epinephrine (either via nebulisation (3 mg) or via metered-dose inhaler and spacer (625 mcg)) given 30 min apart in the ED and two doses of once daily oral dexamethasone (0.6 mg/kg per dose, up to a maximum of 10 mg). Participants will be randomised in a 1:1 ratio to either the placebo or the EpiDex combination therapy. The BIPED study aims to provide the requested additional evidence [35, 36] after a previous study unexpectedly found that EpiDex reduced hospitalisation within 7 days of an ED visit by one-third [33].

### Research ethics approval

The BIPED study was approved by Health Canada and the local research ethics committee at each study site prior to enrollment. The remote elicitation exercise was approved by the Hospital of Sick Children research ethics committee. Implied consent was used for the remote elicitation exercise, meaning that by partaking in the elicitation exercise, the experts agreed that their data could be used for research.

### Designing the remote elicitation exercise

#### Key parameters and clinical setting

The primary outcome in the BIPED study is admission to hospital within 7 days following initial presentation to ED with bronchiolitis, which can be modelled using a binomial distribution. The parameters of interest in the BIPED study are the probability of hospital admission within 7 days for each arm, placebo and EpiDex, denoted *π*_1_ and *π*_2_, respectively. As beta distributions commonly model beliefs about probabilities [37], we assume that each expert’s prior can be expressed as a beta distribution.

To enable the elicitation, we developed a clinical case study (Supplementary Material) of an infant with bronchiolitis, who would meet the inclusion/exclusion criteria of the BIPED study, and was likely equivocal with respect to admission into hospital (i.e., EpiDex could potentially improve infant prognosis if prior beliefs supported benefit). Experts were asked to determine the number of patients, out of 100, with characteristics like this patient who would be admitted to hospital within 7 days under the two different treatment options. Thus, we elicited the two probabilities as proportions among a population of similar patients [38] and felt that 100 patients were sufficient to capture uncertainty in this proportion rather than sampling variability. We elicited the probabilities of hospital admission, rather than a treatment effect, as this more closely reflects everyday clinical decision-making and thus the experts were more able to provide information on these probabilities. However, in doing so, we assume independence between the two admission probabilities, which may be incorrect.

The elicitation exercise aimed to determine prior distributions for the BIPED Bayesian SSD and analysis. However, as placebo is not offered in routine care, we elicited the probability of admission under supportive care. In the BIPED study, placebo will be offered with supportive care (to avoid bias) and, thus, we assumed that the prior for the placebo in our Bayesian SSD was represented by the prior for supportive care.

#### Developing an online elicitation tool

Our remote elicitation exercise used an adapted version of the Sheffield Elicitation Framework (SHELF) methodology [17, 25], which aligns closely with the IDEA protocol [39]. Online tools have supported the use of the SHELF framework [40, 41] and we adapted these tools for our elicitation. We built a Web-based interactive elicitation tool using R software and the shiny package [42, 43] (https://phebelan.shinyapps.io/Elicitation/). In this tool, experts were asked to provide the lower and upper plausible values that subjectively described their beliefs about the number of infants with bronchiolitis who would be hospitalised within 7 days. We assumed that the lower and upper plausible values represented the limits of the 95% central credible interval in the beta distribution. Experts then provided their “Best” estimate for the number of hospitalisations. This best estimate was assumed to be the point of highest probability density in the prior and, thus, represented its mode. Within the online tool, we restricted the value for the mode to be within the plausible interval. By eliciting the upper and lower plausible values, followed by the mode, we aimed to prevent experts from anchoring to their initial most likely value and thereby underestimating uncertainty [24]. The online tool provided experts with a real-time individual beta distribution plot, with parameters estimated using R function, BetaExpert [44], and a quantitative summary of their beliefs so they could adjust if the fitted beta distribution did not represent their beliefs (Supplementary Material).

While the online tool supported the elicitation process, the Research Electronic Data Capture (REDCap) application, a Web-based application designed to support secure data capture for research studies [45, 46], collected the elicited distributions from each expert. Once we developed the online elicitation tool and REDCap database, we piloted our workshop three times internally (AP, SD, TK, MO) to ensure clarity of expression, understanding and acceptability of the tool. These workshops were piloted remotely to ensure seamless delivery and the efficient use of experts’ time.

#### Selecting the experts

We targeted experts from Canada, the USA, Australia and New Zealand to determine representative aggregate priors across the regions in the BIPED study, avoiding selection bias. Participants were eligible for the elicitation workshop if they (i) were identified as experts in bronchiolitis and its treatment and (ii) had experience in paediatric emergency medicine (PEM). Participants were excluded if they had extensive prior involvement with the BIPED study, i.e. serving as a site principal investigator. Potential participants were invited to contribute by email. We aimed to recruit between 10 and 20 experts to ensure a breadth of experience in terms of geography and speciality [14, 47].

#### Determining an aggregate prior distribution

In elicitation, determining an aggregate prior distribution is viewed as a consensus formation process, in which the pooled prior distributions should fairly represent all individuals’ beliefs [48]. In our elicitation study, each expert *i* = 1, …, *N* generates a prior distribution for each trial arm *j* = 1, 2,

$$ {p}_{i,j}\mid {\boldsymbol{x}}_{i,j}\sim Beta\left({\alpha}_{i,j},{\beta}_{i,j}\right), $$

where ${\boldsymbol{x}}_{i,j}=\left({x}_{i,j}^1,{x}_{i,j}^2,{x}_{i,j}^3\right)$ is the lower plausible value, mode and upper plausible value, respectively, from the expert elicitation process. These individual-level distributions are combined using the equal-weighted linear pooling method as it can reduce biases introduced by overoptimism and overconfidence [49]. The equal-weighted linear pooling is also easier to implement than other aggregation methods [50]. However, it can lead to less informative prior distributions [50, 51]. We chose to prioritise the ease of use as the experts were not available for long sessions; the lack of informativeness would be overcome by combining the prior with RCT data.

Thus, the aggregate distributions are equal to

$$ {\pi}_j\sim \frac{1}{N}\ \sum \limits_{i=1}^N{p}_{i,j}\mid {\boldsymbol{x}}_{i,j} $$

and we assume that they represent group’s beliefs on the admission rate of infants with bronchiolitis under supportive care and EpiDex, respectively, for *j* = 1, 2. We generate separate pooled distributions for each region and for each workshop to explore differences.

### The remote, real-time elicitation workshop

#### Pre-workshop materials

One week prior to the workshop, all participants were sent a study dossier to read before attending the workshop. This dossier aimed to introduce the concept of an elicitation exercise and the current literature on treatments for bronchiolitis [24]. Our study dossier included a published elicitation study similar to our study [17] and four manuscripts presenting the use of epinephrine and/or dexamethasone as a treatment for bronchiolitis [33, 52–54]. The previous elicitation study was included to introduce the concept of Bayesian probability distributions and elicitation, while the other studies were included to complement the experts’ knowledge with the current literature.

#### Remote, real-time expert elicitation workshop

We conducted three remote, real-time elicitation workshops using Zoom, a cloud-based video conferencing platform [55], and a standardised script (Supplementary Material) [49]. Three facilitators from the BIPED study team with statistical and medical content specific-expertise attended each workshop (JL, SD, AP, TK, AH). The workshop began with an introduction to Bayesian statistics, probability and the BIPED study. To familiarise experts with the elicitation procedure, an example using our online elicitation tool was then shown (Supplementary Material). Experts then used the online elicitation tool to provide their personal beliefs about the chance of hospitalisation for the patient identified in the case study.

The elicitation exercise was structured over two rounds with a group discussion between the two rounds [24, 25]. In the first round, experts provided their individual prior distribution for the probability of hospitalisation with supportive care and EpiDex. The facilitator (JL) then generated a deidentified boxplot (Fig. S1) to display all the individual-level priors and support the group discussion. The group discussion allowed the experts to adjust and calibrate their responses but did not aim to reach a consensus [24]. The group discussion began with the facilitator interpreting the individual boxplots before the experts were encouraged to share their beliefs and discuss their thoughts around the observed variations in beliefs across experts. When the group discussion no longer resulted in an exchange of information, the facilitator managed the discussion to help promote critical thinking [39]. Following the group discussion, experts were asked to use the online elicitation tool to characterise their beliefs and these results then generated the individual prior distributions to be pooled.

#### Following the remote, real-time elicitation workshop

Following the completion of all three workshops, the experts were sent the pooled distributions for the probability of hospital admission with supportive care and EpiDex. The experts were also sent the workshop-specific pooled distribution for each workshop and their own individual distributions for comparison and were invited to provide comments on the aggregate distribution.

### Bayesian sample size determination

To determine the sample size in the BIPED study, we used the average length criterion (ALC) for Bayesian SSD [56]. This method selects the smallest sample size for which the average length of a specified posterior credible interval is below a given threshold. The ALC uses a preposterior analysis where the length of the posterior credible interval is estimated across the prior-predictive distribution of the potential studies [56]. To achieve this, we simulated the probability of hospitalisation within 7 days under the two treatments based on the priors from the expert elicitation exercises using a binomial likelihood. These simulated data were combined with our aggregated prior to determine the posterior for the two probabilities of hospitalisation, using Markov chain Monte Carlo (MCMC) methods [57]. We then calculated the 95% high-density posterior credible interval for the difference in the probability of admission across the two treatments, placebo and EpiDex. We estimated the average posterior credible interval length for sample sizes between 400 and 630 using 1500 simulations from the prior-predictive distribution and 5000 simulations from the posterior. We selected the sample size for which the ALC is below 0.09.

In the BIPED study, we will declare that EpiDex is superior to placebo if the posterior probability that the probability of hospitalisation under EpiDex is greater than the probability of hospitalisation under placebo exceeds 0.99;

$$ P\left({\pi}_1<{\pi}_2\right)>0.99. $$

To determine whether our study is likely to reach a conclusion, we compute the relative predictive power of the study, defined as the probability of declaring EpiDex is superior based on simulated studies from the prior predicative distribution, standardised by the prior probability that EpiDex is superior to placebo. These calculations were based on 8000 simulated trials with 5000 simulations from the posterior. All Bayesian analyses were performed using JAGS through R [43, 58].

## Results

### Elicitation workshop

#### Baseline characteristics

We invited 25 PEM clinicians from Canada, the USA, Australia and New Zealand to participate in our three remote elicitation workshops. Fifteen of these experts agreed to participate in the study: 9 from North America (NA) and 6 from Australia and New Zealand (ANZ). The three workshops contained 5 (2 NA; 1 ANZ), 4 (4 NA) and 6 (3 NA; 3 ANZ) participants, respectively. Table 1 displays the baseline characteristics for these 15 experts. Experts from NA had more experience treating bronchiolitis with epinephrine and dexamethasone, separately and combined. However, most experts do not currently use either treatment in their routine practice.

**Table 1:** Baseline characteristics of experts, by region of practice

Treatment	All	North America	Australasia
Number of responses	15	9	6
Has experience treating bronchiolitis patients with: *n* (%)			
Epinephrine	10 (67)	9 (100)	1 (17)
Dexamethasone	2 (14)	1 (12)	1 (17)
Epinephrine and dexamethasone	5 (34)	4 (45)	1 (17)
Currently treating bronchiolitis patients with: *n* (%)			
Epinephrine	4 (27)	4 (45)	0 (0)
Dexamethasone	0 (0)	0 (0)	0 (0)
Epinephrine and dexamethasone	0 (0)	0 (0)	0 (0)

#### Prior distributions

**Fig. 1:**
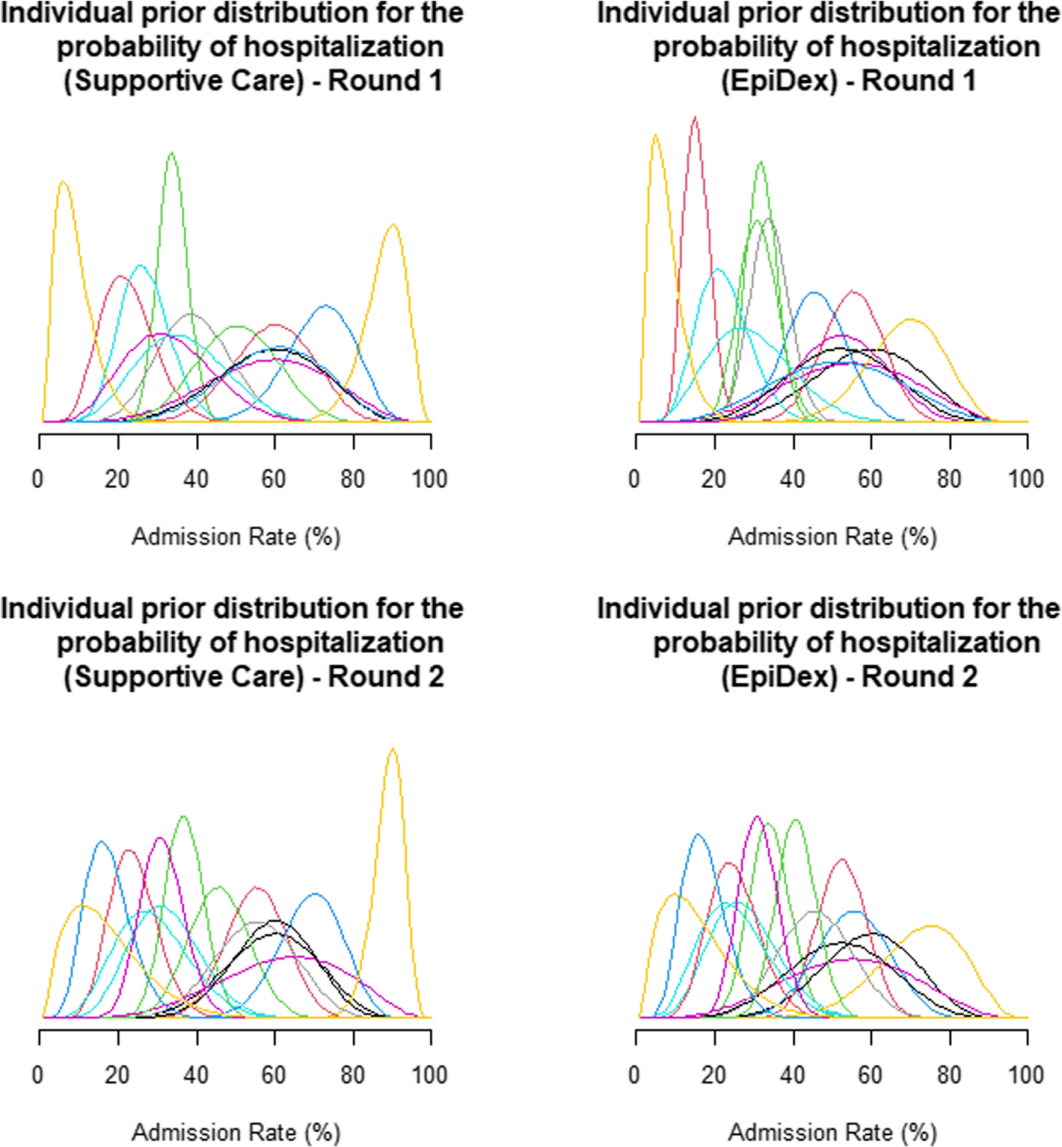
Individual-level elicited prior distributions for hospitalisation probability under (**a**) supportive care (left) or (**b**) treatment with the combination of epinephrine and dexamethasone (EpiDex, right). Each line depicts the distribution scored by an individual participant (*n* = 15). Distributions for first elicitation round on top; second round at bottom

**Fig. 2:**
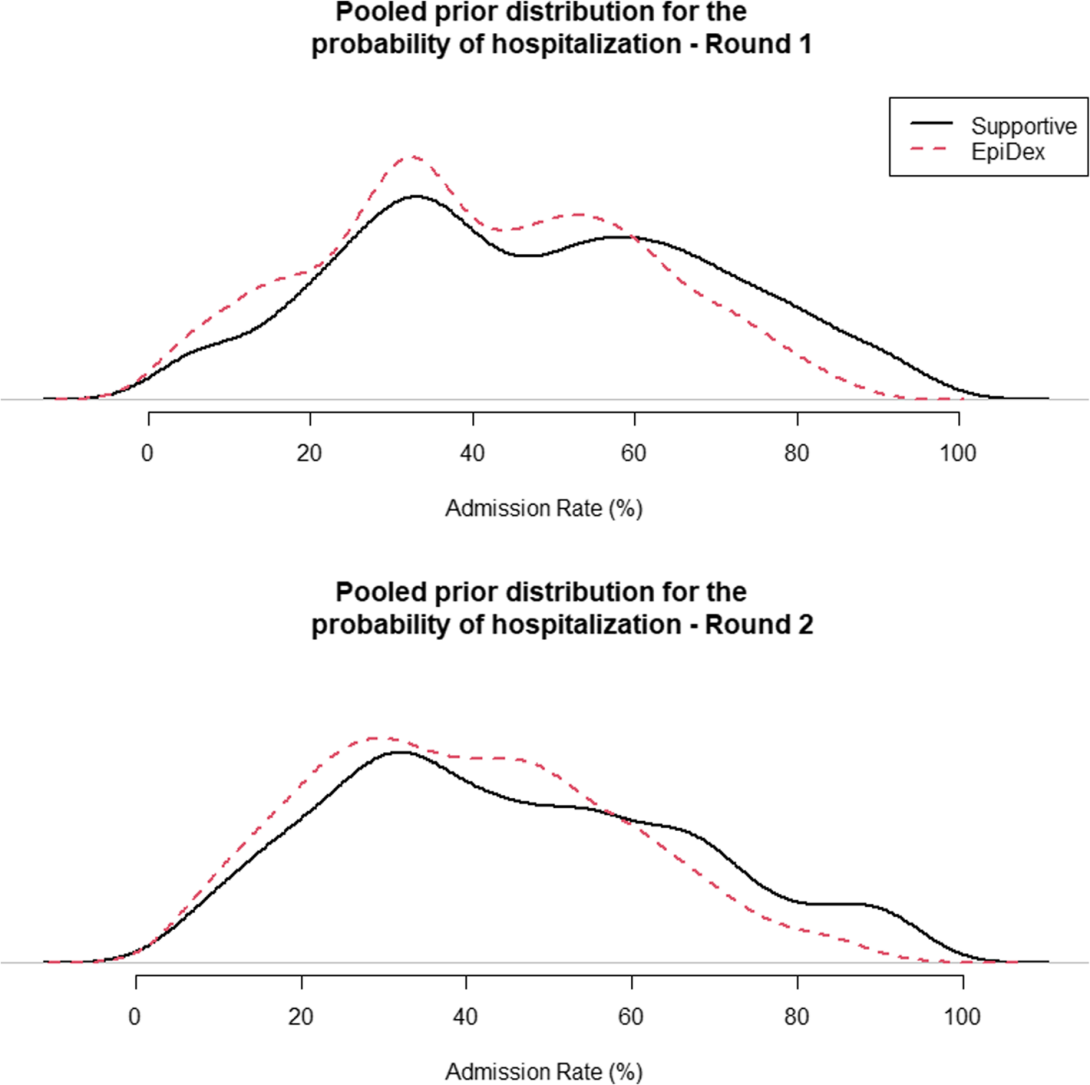
Pooled elicited prior distributions for hospitalisation probability under (**a**) supportive care (supportive, solid black line) or (**b**) treatment with the combination of epinephrine and dexamethasone (EpiDex, dashed red line). Distributions for first elicitation round top; second round bottom

Figure 1 displays the individual prior distributions for the two probabilities of hospitalisation, with supportive care on the left and EpiDex on the right. The individual responses from both rounds were highly varied, both in terms of the central tendency of the distributions across individuals and the width of the plausible interval within individuals, although most experts believe that the probability of admission for infants with bronchiolitis is slightly lower for those receiving EpiDex compared to supportive care. Figure 2 displays the pooled prior distributions from all experts for each round in the elicitation workshop. In both rounds, the pooled prior distributions show a slight reduction in the probability of admission for infants with bronchiolitis who are treated with EpiDex. However, experts were less certain about the size of this reduction in the second round, demonstrating that the group discussion led the experts to be more conservative. These distributions are multi-modal, which is a result of the equal-weights linear pooling method. Thus, they do not represent a consensus distribution for a single rational impartial observer but rather acknowledge that there are differences in beliefs across the experts [50]. No experts provided critiques on these priors by email.

Pooled prior distributions demonstrate that similar beliefs about the probability of hospitalisation are held in NA and ANZ (Fig. S2) but that the aggregate distributions were different for each workshop (Fig. S3). However, this is likely due to the diversity of our experts and the limited number of individuals in each workshop.

### Bayesian sample size determination

**Fig. 3:**
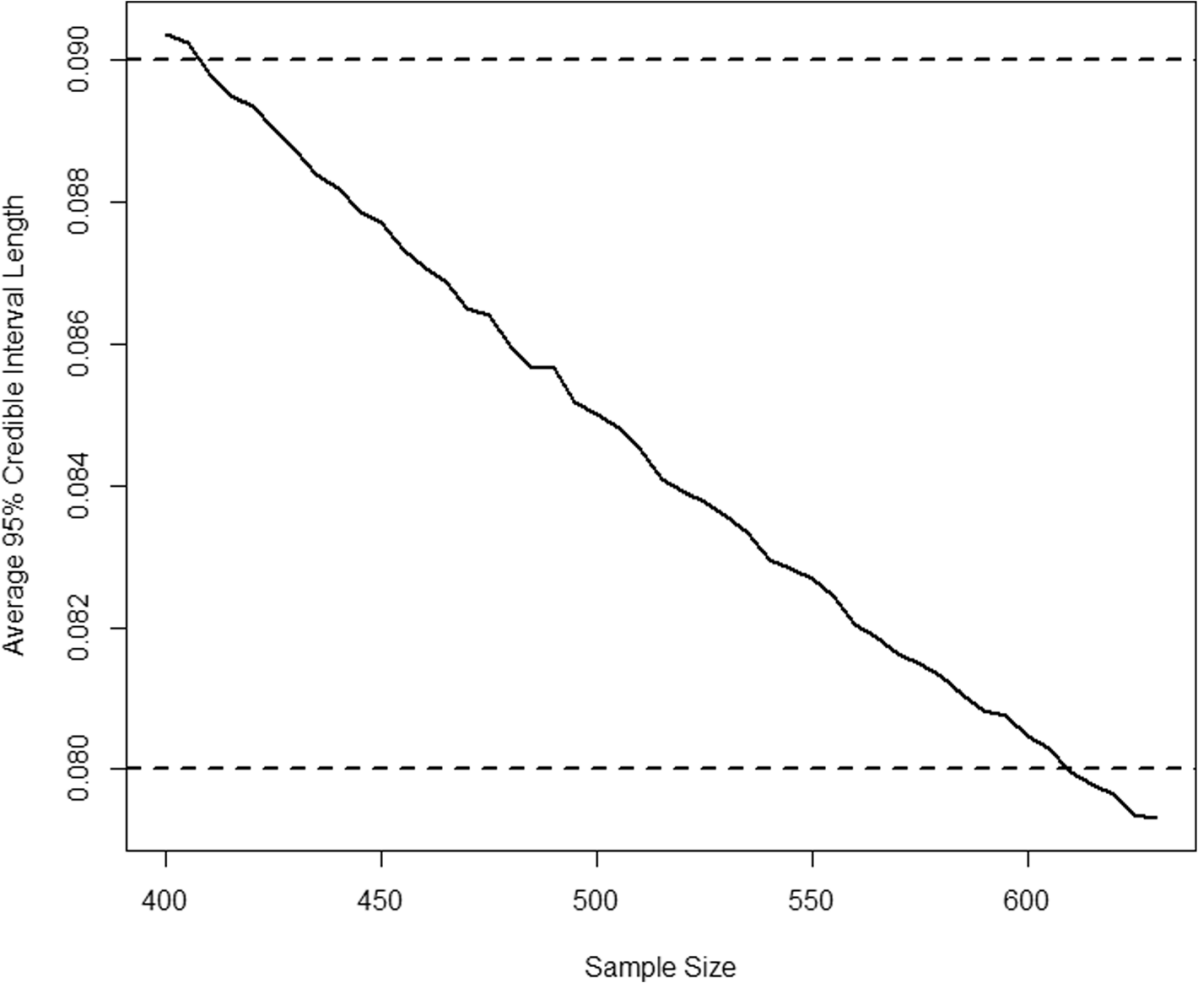
The width of the average 95% posterior credible interval length for “admission probability difference” between placebo and the combination of epinephrine and dexamethasone EpiDex plotted across the BIPED clinical trial sample sizes increasing between 400 and 630 in increments of 5 (solid black line). Average length criterion (ALC) thresholds of 0.09 and 0.08 are plotted as dashed black lines (see text)

We computed the average length of the 95% high-density posterior credible interval for the difference in admission probability between the two arms (Fig. 3). From these results, we specify a sample size of 410 participants per arm for the BIPED study ensuring the average 95% credible interval is shorter than 9%, compared to 610 participants per arm with a uniform prior. Adjusting for an expected 5% loss to follow up, the total sample size of the BIPED study is 432 per arm. The average 95% credible interval would be less than 8% if the BIPED study recruits 610 participants per arm. With 410 participants per arm, the study has a relative predictive power of 90%.

## Discussion

We implemented a remote, real-time elicitation exercise that provides a practical and convenient method for expert elicitation using an online tool. Expert belief, elicited using this framework, then formed the basis of a Bayesian SSD and analysis for an international RCT, where the prior distribution should represent the diverse beliefs across the regions enrolling patients. The remote nature of our elicitation allowed us to practically obtain diverse opinions by running a synchronous online exercise with a reasonably large number of diverse experts. We quickly enrolled 15 experts from 4 countries (Canada, USA, New Zealand and Australia), on a limited budget, under COVID-19-related travel restrictions and determined a pooled prior distribution that represents the diversity of perspectives in an international trial. As our elicitation exercise involved a relatively short time commitment, we had high response rates, resolving issues seen with asynchronous elicitation [22]. Finally, we were able to hold multiple elicitation workshops assisted by a facilitator to further broaden the range of experts who could attend.

Another advantage of our elicitation framework, compared to asynchronous elicitation methods, is that we were able to have real-time facilitation and a group discussion [21]. This allowed us the interaction between experts and the identification of issues within the workshops. As there were differences between the distributions between the two rounds, the group discussion was critical in calibrating the experts’ beliefs. The experts raised external factors that would influence the decision to admit an infant with bronchiolitis, such as hospital resources and family circumstances, and shared their thoughts and clarifications related to the design of the elicitation exercise and their understanding of the elicitation task. We were also able to respond to any technical issues and ensure that all enrolled experts were able to provide responses.

Our biggest challenge was scheduling the workshops as we needed to accommodate large differences in time zones between the countries and the shift patterns of practising PEM clinicians working in the ED. We decided to run multiple workshops so more experts could participate and aimed to include experts from each region in each workshop and ensure there were enough participants to allow a fruitful group discussion. While we were largely successful, we found that scheduling of these workshops was a significant challenge and recommend inviting a higher number of experts than required as some schedules may be incompatible, especially across multiple time zones. Furthermore, scheduling multiple workshops prevented us from using expert consensus to determine the final prior distribution. Instead, we used the equal-weights linear pooling method, which has been found to provide less informative prior distributions [50].

A strength and limitation of our remote, real-time elicitation exercise is the time taken for the workshop. Each workshop was scheduled for 90 min, and the experts were invited to read five manuscripts before attending the workshop as preparation — estimated to take another 90 min. This minimal time commitment, compared to day-long meetings and travel, allowed us to recruit a range of experts to our study and was key to enrolling practising PEM physicians. However, the set 90 min meeting-slot did limit the time available for presenting the theory behind elicitation and for calibration exercises, which could have impacted the quality of our elicited prior distribution [38] and prevented us from using alternative pooling methods [59]. We also did not consider multivariate elicitation [60], which would have allowed to consider dependence between the two admission probabilities.

## Conclusions

To enable application of Bayesian methods for SSD, we implemented a remote, real-time elicitation exercise that offers a comprehensive, practical, affordable approach to obtaining prior distributions for a Bayesian analysis of an international RCT. This prior distribution was then used to determine the sample size for the proposed Bayesian analysis. Thus, this remote, real-time elicitation exercise can promote the use of Bayesian methods in randomised controlled trials.

## Supplementary Information

**Additional file 1:** Supplementary material

## Data Availability

Our consent procedures do not allow for data sharing due to the small number of participants in the study and the ease of identifying these individuals. The pooled distributional forms are available from the corresponding author on reasonable request.

## Abbreviations

ANZ: Australia and New Zealand
BIPED: Bronchiolitis in Infants Placebo Versus Dexamethasone Study
REDCap: Research Electronic Data CAPture
EpiDex: Combination therapy of nebulised epinephrine and dexamethasone
ED: Emergency department
NA: North America
PEM: Paediatric emergency medicine
