# Supplementary material for: Remote, real-time expert elicitation to determine the prior probability distribution for Bayesian sample size determination in international randomised controlled trials: Bronchiolitis in Infants Placebo Versus Epinephrine and Dexamethasone (BIPED) study
Source: Trials. 2022 Apr 11;23:279. doi: 10.1186/s13063-022-06240-w (PMC8996198; doi:10.1186/s13063-022-06240-w)
Supplement: Supplementary file 1 — Additional file 1: Supplementary material [file 13063_2022_6240_MOESM1_ESM.docx]

**Remote, real-time expert elicitation to determine the prior probability distribution for Bayesian sample size determination in international randomized controlled trials: Application to the Bronchiolitis in Infants Placebo Versus Epinephrine and Dexamethasone (BIPED) Study**

***Supplementary Material***

1. *Clinical Case Study for Elicitation*

Consider a 6 month old presenting in the winter months to the ED. His symptoms started 2 days ago with a runny nose and mild cough. One day ago, he developed a fever (up to 39 C rectally) and a worsening cough. Today his mother notes he is having a harder time latching onto the breast, taking about 50-75% of usual volumes, has had two wet diapers since waking at 7 am (it is now 3 pm), and seems to be having a hard time to breath. On physical examination his oxygen saturation is 93%, temperature is 38 C rectally, heart rate is 150 bpm, respiratory rate is 55 bpm, he has mild to moderate intercostal and subcostal indrawing and diffuse inspiratory and expiratory wheezes. You diagnosis him with bronchiolitis.

1. *Screenshot of Web Interface*


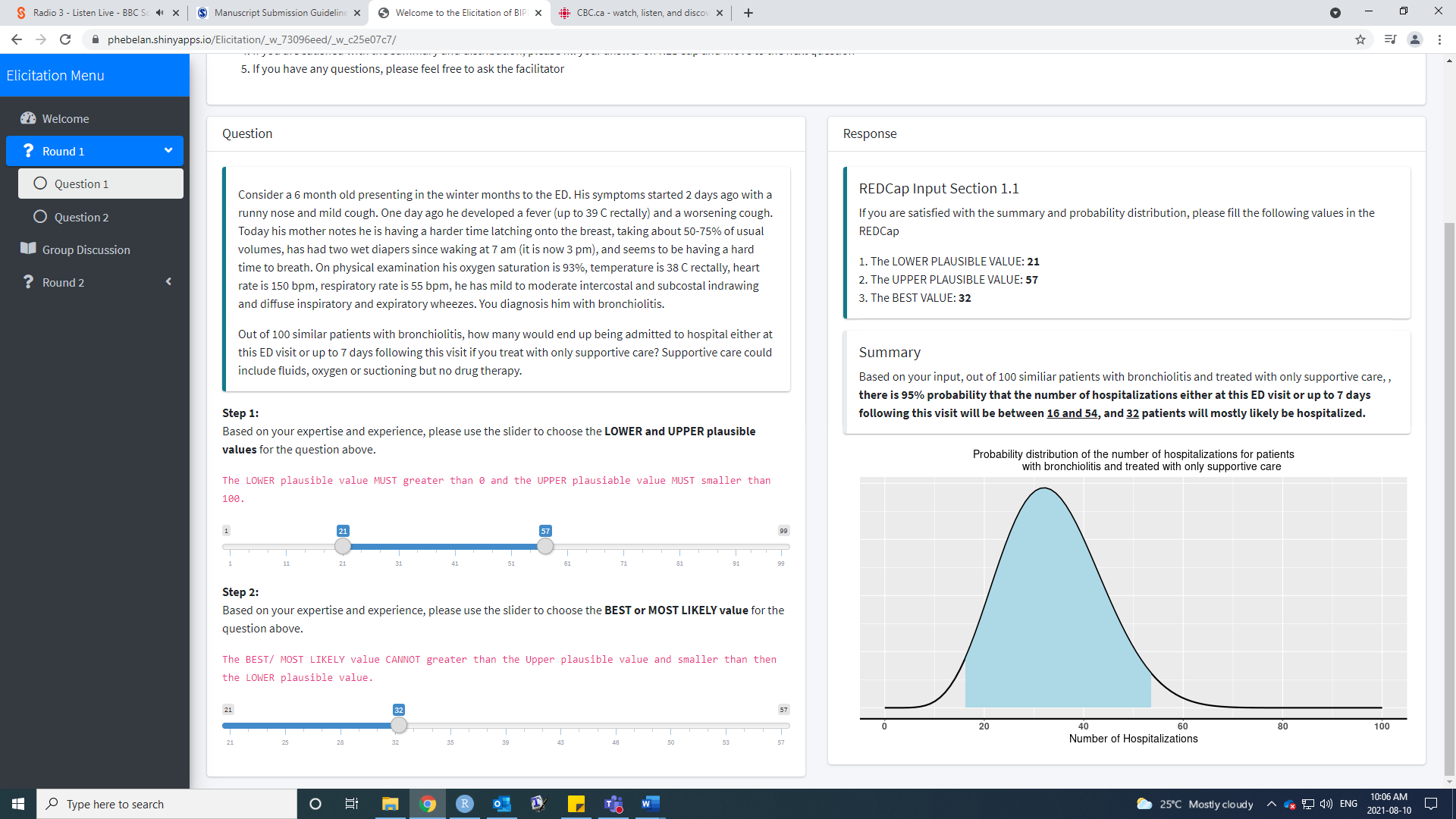


1. *De-identified boxplot to facilitate group discussion*


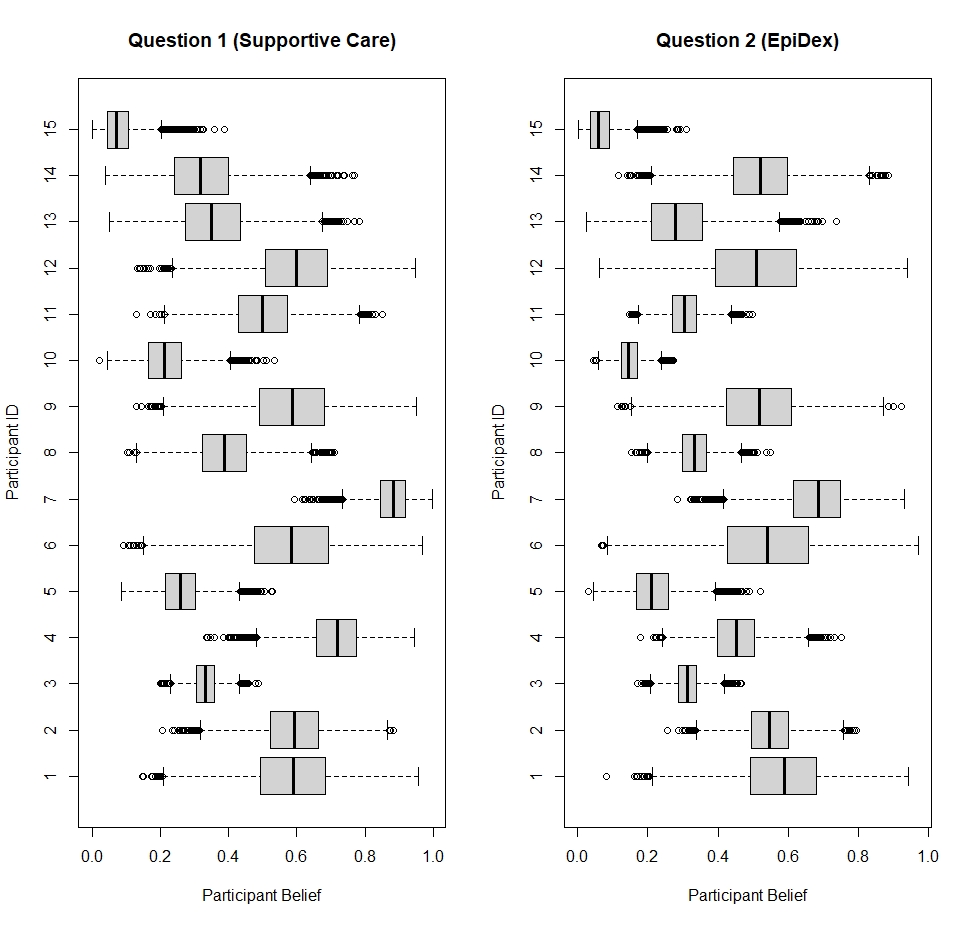


*Figure S1: Boxplots displaying the individual elicited distributions from the first round of elicitation for hospitalisation probability under a) supportive care (left), or b) treatment with the combination of epinephrine and dexamethasone (EpiDex, right). Participants are numbered by their enrolment time in the BIPED elicitation study. These boxplots were displayed during the elicitation exercise to facilitate the group discussion, with each workshop only displaying and discussing the individual elicited distributions from experts participating in that workshop.*

1. *Elicited prior distributions by workshop*


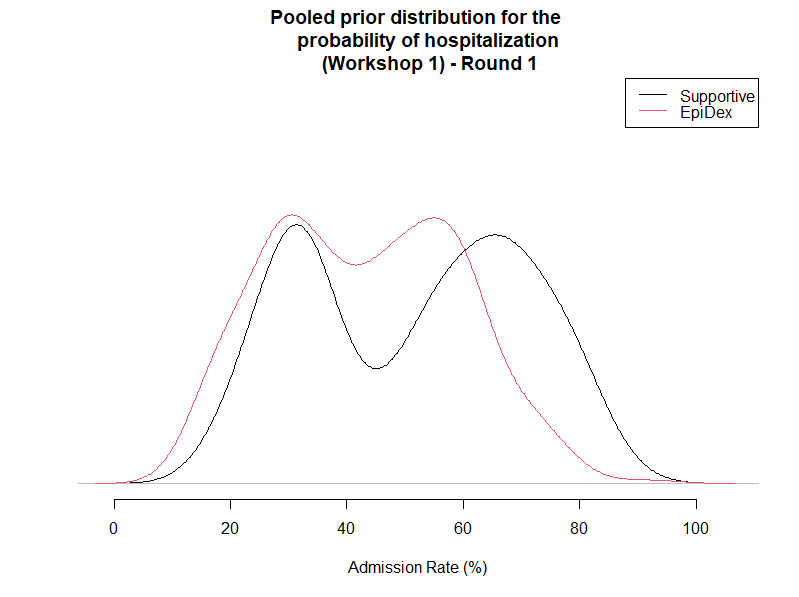

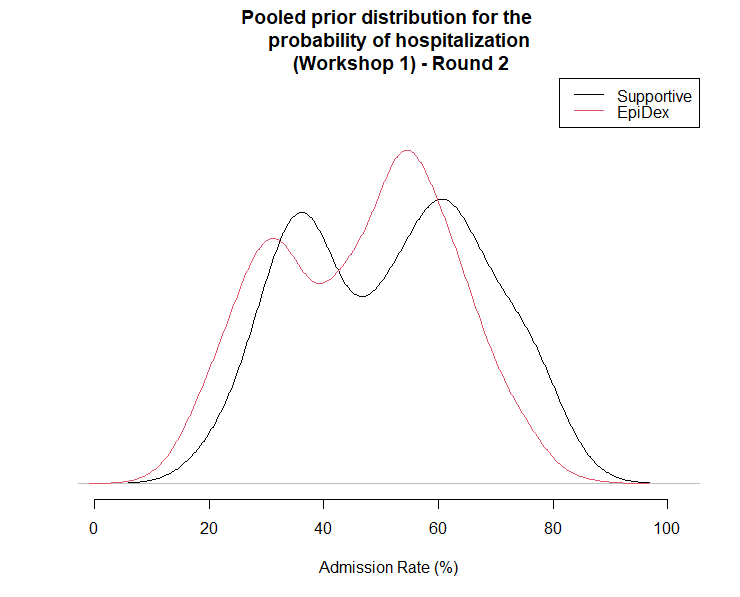

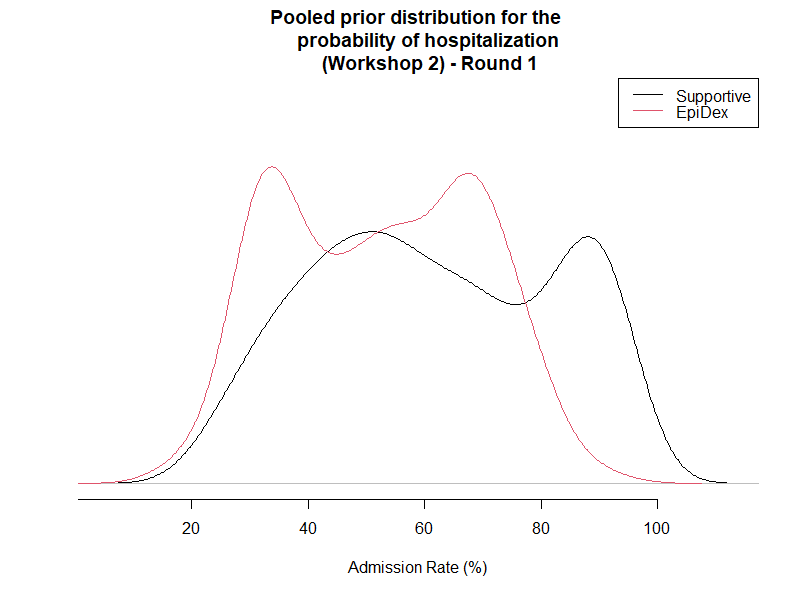

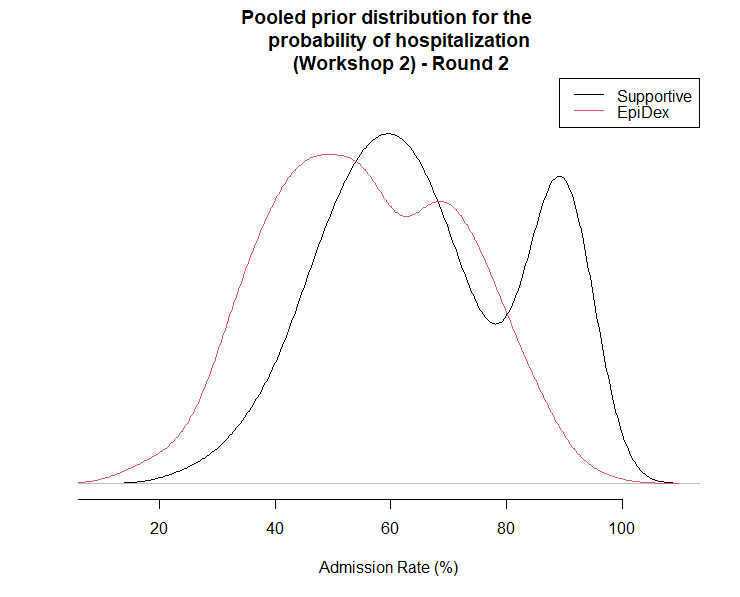

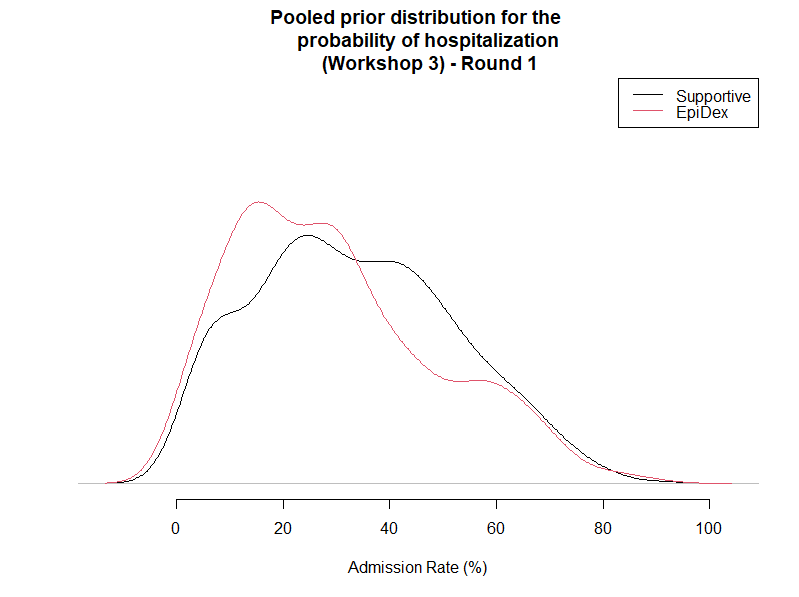

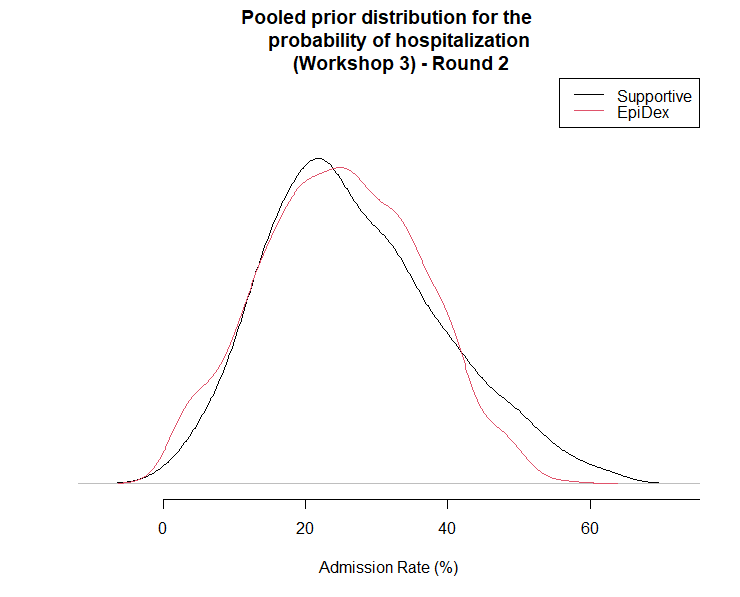


*Figure S2: Pooled elicited prior distributions for hospitalisation probability under a) supportive care (black line), or b) treatment with the combination of epinephrine and dexamethasone (EpiDex, red line). Distributions for first elicitation round left; second round right. The top two plots display the distribution from the first elicitation workshop, the middle two from the second elicitation workshop and the bottom two from the third workshop.*

1. *Elicited prior distributions by expert practice region*


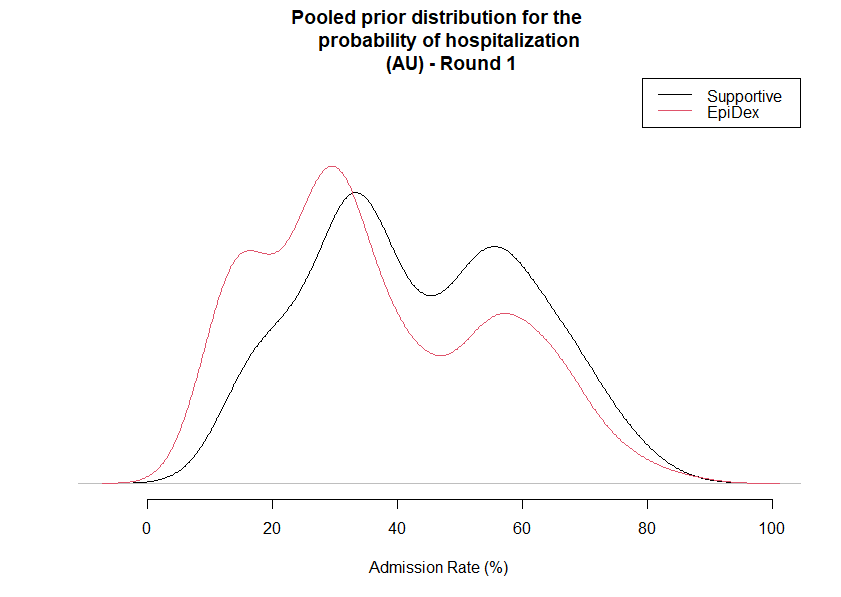

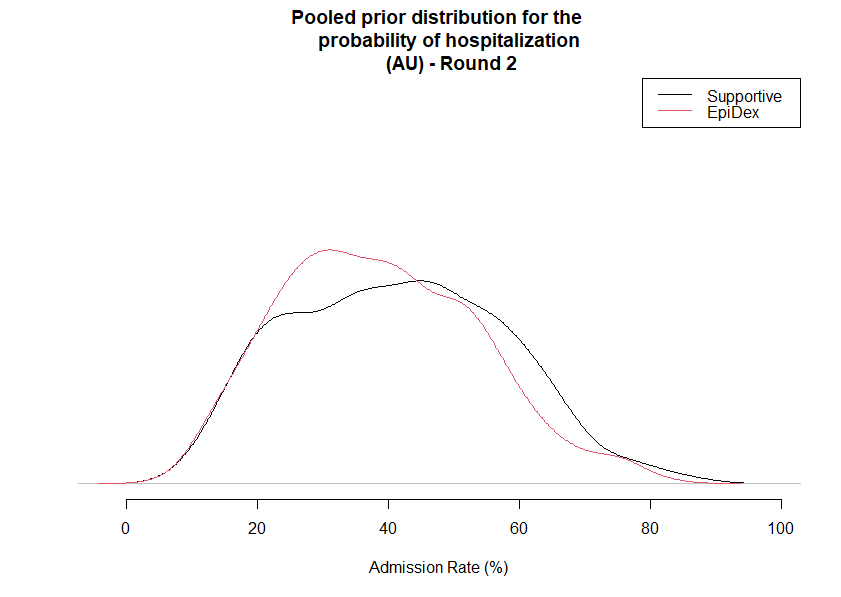

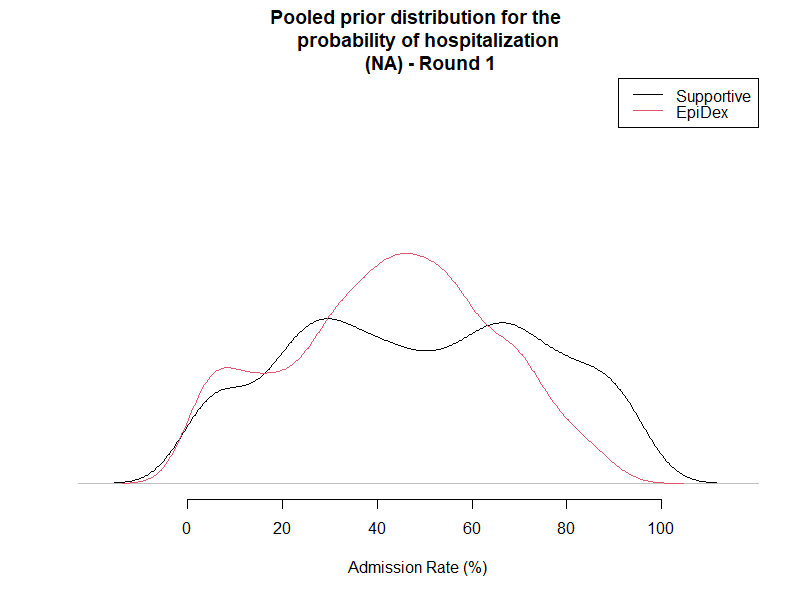

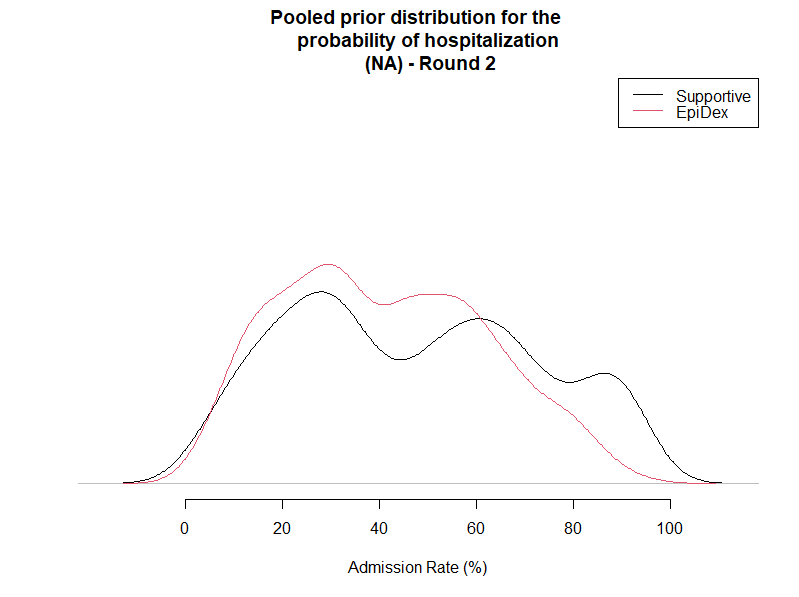


*Figure S3: Pooled elicited prior distributions for hospitalisation probability under a) supportive care (black line), or b) treatment with the combination of epinephrine and dexamethasone (EpiDex, red line). Experts who practice in Australia and New Zealand (AU) on top; in North America (NA) bottom.*

1. *Script for the elicitation workshop – page numbers refer to slide numbers below:*

**Introduction**

Hello everyone, welcome to the elicitation workshop for the BIPED study.

[X], thank you for the introduction. I am Phebe, the one email to you all the time, I am the facilitator for the workshop today. Actually, we are running three workshops in total in order to have as much experts as possible, and don’t worry, everyone only needs to join the workshop once. Today is our X workshop and I would like to give a brief introduction on how it will go.

**Page 2**

The BIPED study is a phase III multicenter, randomized, double-blind trial comparing treatment with epinephrine and dexamethasone to the supportive care for infants with bronchiolitis. It aims to determine if treatment of infants presenting with bronchiolitis to the emergency department with epinephrine and dexamethasone is effective in reducing the need for admission to hospital either at this ED visit or up to 7 days following this visit compared to only the supportive care. The purpose for this workshop is to formulate a prior probability distribution that can be further used to calculate the sample size for BIPED study using Bayesian analysis.

**Page 3**

I believe most of you have heard or used frequentist sample size calculation, which is seeking for the smallest sample size that is sufficient to achieve a desired power at a significance level. It turns out we need to make a lot of assumptions like the power, effect size and significance level. Bayesian statistics is different with frequentist statistics. We do not need to make a lot of assumptions and we will use a prior distribution which is your belief about how likely it is that a current event will occur. So, each of your answer which is based on your expertise and past experience will be used to generate an individual probability, and of course, everyone has different judgements, so there is a subjective level of uncertainty and can vary among you guys. We will aggregate your individual distribution to form a prior distribution, and hopefully, this distribution can represent all your opinion or judgements on the questions in the questionnaire we are going to complete in today’s workshop.

**Page Consent**

Before jumping into our workshop, I would like to let you know this questionnaire involves minimal risk to you. In terms of the benefits, you will gain more knowledge in the treatment of bronchiolitis and will be provided with study results to see the quantitative expression of your subjective judgement. We hope that the information learned from this study can be used in the future to benefit other researchers who are aiming to understand how the current bronchiolitis treatments are viewed by experts. The output from this study will also be used to support the analysis of the BIPED study once it has been completed, which will support the treatment of infants with bronchiolitis.

If you have any questions about this project, you may contact me or Amy or Stuart or Terry, we are very happy to provide any additional information. By completing these questionnaires, you are consenting to its use in research.

All information collected about you will be “de-identified” by replacing your identifiable information (i.e., name) with a “study number”. Only the “study code key” can connect the information collected about you to your identity. The study code key will be safeguarded by the SickKids research team and will not be available to other agents. Even though the risk of identifying you from the study data is very small, it can never be completely eliminated.

**Page 4**

Today, we have X experts from North America and Australasia, thank you so much for your time and we really appreciate your participation. How about let’s have a short introduction, I will just go the order on my side.

Perfect! Hope everyone could enjoy the workshop today.

The workshop today will have three survey links and the first one is the personal information or personal experience. I already sent the email with the link 15 minutes before the workshop. If you haven’t received it yet, please just let me know. Other than this, we will have two rounds of questionnaires which have two questions each round. You will have up to 8 minutes to complete the questionnaire using Rshiny dashboard and enter your answer in the REDCap. I will show you how to do this in a moment. We want to have three values the lower plausible value, upper plausible value, and your best guess value from each question. So, in total it would be 6 values from you each round. On the Rshiny Dashboard, all the questions and helpers are on that page. There is a real-time summary and distribution plot on the question page to help you change your values or estimates until the summary and distribution matched your opinion.

**Page 5**

After you completed the link in the email, I already sent to you, you will receive the second email immediately. In that email, you will see a REDCap link, so by click that link, you will see a page like this picture. This is the place that you need to enter your answer manually in order to saving your data securely. You may curious there is no question on this page.

**Page 6**

Good catch, in that second email, there is also a Rshiny dashboard link, so you need to click that link too, which means you need to have two websites opened. One is for reading the questions and find the answer, another which is the REDCap is the place that will store your answer.

**Page 7**

On the Rshiny page, you can see there is a round 1 button, by clicking that, there is question 1, and this is the starting point for you.

**Page 8**

Once you get to the round 1 question 1 page, you will have something looks like this page. Don’t worry, you do not need to read the words in the red box, we will show you the details later. So once you reach this page, first step is to read the instructions, then you can read the questions, and use the slider in the box 3 here to choose the lower and upper plausible value. After that, you can use the slider in the box 4 to choose the best guess value. Once you have all the numbers, you need to check out the summary and plot in the box 5. You may find out the number in the summary is slightly different with the slider’s values. Since we are using some statistics behind it by assuming a beta distribution. This is fine, you could just change the slider value, until the summary and plot matched your opinion. After that is the most important part, please enter the value in the REDCap Input Section in the REDCap page, which is the survey link. As I mentioned before, the summary would be slightly different with your sliders value, so please enter the slider value which is box 6 in the REDCap.

**Page 9**

Now let me show you an example. As you can see, I have two tab or websites opened. This is the REDCap, and this is the Shiny page.

I will read the instructions first, then the question. In Canadian universities, most of students are using windows and MacBook. Assume we have 100 participants, how many of them using a windows laptop. So, step 1 is to choose the lower and upper plausible value of the number of people you think they use a windows laptop? Based on my experience in school, I have seen most of them using a MacBook in the courses I have taken. Therefore, I believe the lower plausible value for the number students would be 20 out of 100. However, some of the computer science courses, students must use a windows laptop because of some programming languages. In this case, I think the upper plausible value would become 40 out of 100. Once I have determined these two values, I can consider what the most likely value of the number of students using windows laptop in Canadian university. Since there are a lot of disciplines at school, so I believe the most likely value would closer to the lower plausible value, and I think 28 makes sense to me. So, after that, I look on the right side, I can see the distribution and the summary, if you think it’s matched with your opinion. Great, then enter the value in the upper box into the REDCap.

**Page 10**

So once the formal elicitation process starts, you can click the round 1, and you can see questions here, which will have a very similar format with the example questions. After everyone completing the questionnaire, there will be a short break, and I will generate a deidentified boxplot that have everyone’s answer, then we will have a 10–15-minute group discussion. Please share your thoughts with other group members, but remember it is not necessary to reach a consensus among these questions, it just helps you to calibrate your judgements. So, in the second questionnaire, you may adjust your values feel more confident with your answers. The last and the most important thing again is don’t forget to enter your answer in the REDCap.

**Page 11**

OK, now you are moving to the personal information/ experience step. You may click the link in your email. Once you completed the survey, please send me a message no matter in public chat box or private chat. You could just send 1 to me so I could know how many people finished at the moment. Also, please let me know if you have any questions. And you may click the link now and fill the blanks now.

**Page 12**

It seems like everyone is done with the first link; we will begin the formal elicitation now. You should receive the email named E-BIPED step 2 first round of questionnaire in your mailbox. If you haven’t received it, it may be in your junk box or just let me know. OK, you may click the REDCap Link and Shiny Link in the second email, please make sure you have two windows opened. and click the round 1 question 1 button, hope we all on the same page right now. And you may start to do the first-round questionnaire, please read the instructions and enter the slider number which showed in the redcap input section. Let me know if you have any questions.

We want to have 3 values from you for each question so in total it would be 6 values in round 1. Please make sure the number you entered in the redcap is correct.

Don’t forget to send me a message when you are done and take a short break.

**Page 13**

We are now moving to the discussion stage. In case some of you are not very familiar with boxplots, I have a picture here, it is a horizontal boxplot. So, the box here which is the blue part is 50% probability that you believe the number of patients are under the corresponding treatment arm. The two lines or tails is called whiskers, it represents the ranges for the bottom 25% and upper 25% probability, so if you have a very long whisker, you may have a conservative opinion.

I have shared the boxplot that has everyone’s answer on the screen. CONTINUE TO DISCUSSION.

**Page 14**

Okay, I think we all have some take home messages from the discussion. Let’s move to our last step here the second-round questionnaire. You should have the email named E-BIPED step 3 second round of questionnaire in your mailbox. Please click the redcap link through that email. The process is the same as the first round, and the Rshiny page maybe disconnected, you just need to refresh the page, or if you close the Rshiny page before, you can access it through the link in the email.

Please let me know if you have any questions, and you may start the second round now.

Same as first round, we want to have 3 values from you for each question, and in total it would be 6 values. Please make sure you enter the slider value in the redcap and make sure the value is correct.

Don’t forget to send me a message when you are done.

**Page 15**

Perfect, everyone is done with the questionnaire. Since our last workshop is on June 15th or 16th Australasia time. I will send you an individual and group distribution one week after that workshop, also whether you would like to be acknowledged in the final publication. If you have any questions, please feel free to email me or Amy or Stuart.

In the end, thank you so much for coming and hope you have a great morning, afternoon and evening.
